# Comparative analysis of m6A mRNA modification in *de novo* and established genes during yeast sporulation

**DOI:** 10.3389/fgene.2026.1856601

**Published:** 2026-07-29

**Authors:** Azka Saleem, Yingshan Jiang, Jian Xue, Yuxuan Cui, Chuli Zhou, Fu Liu, Yuan Liu, Yubin Yan, Ruolin Yang

**Affiliations:** College of Life Sciences, Northwest A&F University, Yangling, Shaanxi, China

**Keywords:** epitranscriptomic regulation, evolutionarily young genes, meiotic time-course, N6-methyladenosine, protein-coding genes, *Saccharomyces cerevisiae*

## Abstract

*De novo* genes, a subset of young and lineage-restricted genes arising from ancestrally non-genic sequences, serve as a reservoir for evolutionary innovation. Despite their potential significance, the regulatory mechanisms governing *de novo* genes remain largely unexplored, particularly regarding post-transcriptional modifications. While N6-methyladenosine (m6A) mRNA modification has been extensively studied in epitranscriptomics, its role and dynamics in *de novo* genes compared to established protein-coding genes have not been investigated. This study investigates the m6A epitranscriptome in *Saccharomyces cerevisiae*, examining modification landscapes, peak quantification, position, consensus motif, and temporal dynamics in a curated collection of *de novo* genes using high-resolution m6A-seq2 data. We analyzed the relationships between m6A modifications and gene characteristics including transcript length, expression levels, and evolutionary age during sporulation. To establish connections between differential gene expression and m6A modification patterns, we performed conjoint analyses of differential m6A modification and RNA expression profiles to elucidate the regulatory role of m6A in gene expression control. Functional annotation of key genes was conducted to provide biological context for our findings. Our results reveal that *de novo* genes exhibit a lower apparent per gene m6A burden than established protein coding genes, characterized by fewer modification peaks per transcript, lower peak intensities, and reduced m6A motif density. However, this difference is primarily explained by the shorter transcript lengths and lower GAC motif densities characteristic of DNGs, rather than by evolutionary age itself. Consistent with this interpretation, evolutionary age was not a significant independent predictor of m6A modification after controlling for transcript length and other variables in multivariable analysis. Notably, DNGs display parallel temporal modification patterns across sporulation stages, indicating that they are subject to the same stage-specific m6A dynamics as established genes.

## 1 Introduction

The emergence of *de novo* genes (DNGs) from previously non-coding DNA sequences represents a remarkable facet of genetic innovation and evolutionary adaptability (Van Oss and Carvunis, 2019; Zhao et al., 2024). These genes, which lack homologs in other species, exhibit unique structural and functional properties, providing valuable insights into genome evolution and the development of species-specific traits (Cai et al., 2008; McLysaght and Hurst, 2016; Vakirlis et al., 2018). Despite their potential significance, the regulatory mechanisms and functional roles of *de novo* genes remain largely unexplored, particularly in the context of post-transcriptional modifications such as N6-methyladenosine (m6A).

Chemical modifications of RNA, collectively termed the “epitranscriptome”, play pivotal roles in numerous biological processes. Among these, m6A stands out as the most abundant internal modification found in messenger RNA (mRNA) (Sendinc and Shi, 2023). m6A involves reversible methylation at the N6 position of adenosine residues, catalyzed by methyltransferase complexes (Duan and Cao, 2025). m6A serves as a key factor in regulating RNA metabolism, including splicing, stability, translation, and localization (Desrosiers et al., 1974; Dubin and Taylor, 1975). Recent advances in transcriptome-wide mapping techniques have revealed the abundance and dynamicity of m6A modifications, occurring across thousands of transcripts, with preferential enrichment near stop codons and within 3′untranslated regions (UTRs), coding sequences, and 5′UTRs (Meyer et al., 2012; Shen et al., 2019). The m6A landscape is shaped by a complex interplay of ‘writers’, ‘readers’, and ‘erasers’, ensuring a reversible and adaptable modification profile. In yeast, the methyltransferase complex (MTC) acts as the primary ‘writer’, depositing m6A marks onto target transcripts. Initially described to include Ime4, Mum2, and Slz1, recent studies have expanded the complex to comprise six subunits: Ime4, Kar4, Mum2, Vir1, Slz1, and Dyn2. Five of these subunits (Ime4/METTL3, Kar4/METTL14, Mum2/WTAP, Vir1/VIRMA, and potentially Slz1/ZC3H13) have orthologues in mammalian MTCs, and all except Dyn2 are essential for m6A deposition (Ping et al., 2014; Ensinck et al., 2023; Park et al., 2023a, Park et al., 2023b). Meanwhile, YTH domain family proteins (notably Pho92) serve as ‘readers’, recognizing m6A-modified RNA and mediating downstream effects on RNA metabolism (Schwartz et al., 2013; Ping et al., 2014). Notably, no ‘eraser’ enzyme has been identified in yeast to remove these modifications.

The functional impact of m6A modifications extends beyond RNA metabolism, influencing diverse biological processes, including cell differentiation, stress response, and developmental timing (Roundtree et al., 2017). In yeast, m6A is deposited primarily during early meiosis, the only life stage with extensive m6A modifications. Furthermore, m6A has been implicated in regulating yeast meiosis and sporulation, key processes crucial for the organism’s life cycle and adaptability (Albihlal et al., 2024). Notably, the sequence context of m6A modifications varies among species, with yeast predominantly exhibiting methylation at the GAC motif, distinct from the RRACH motif observed in mammals (Schwartz et al., 2013; Shen et al., 2019). This evolutionary divergence in RNA modification mechanisms underscores the importance of species-specific investigations. While previous studies have explored histone modifications in DNGs (Li et al., 2016), the role of m6A RNA modifications in regulating and shaping the function of these genes remains largely unexplored. This study aims to characterize the m6A modification landscape in *Saccharomyces cerevisiae* transcripts, focusing on newly emerged DNGs and examining how m6A patterns in these genes compare to established protein coding genes (PCGs) during sporulation.

To address these objectives, we conducted a comprehensive bioinformatics analysis, leveraging high-throughput m6A-seq2 and transcriptomic datasets generated during yeast sporulation. We measured and compared m6A levels, peak density, and motif distribution across multiple sporulation stages. This approach allowed us to discern m6A trends in both newly emerged and established genes, examine m6A levels across different age groups to explore potential evolutionary trends, and investigate differential gene expression and m6A methylation to understand their influence on gene regulation and function. Our findings highlight specific DNGs with dynamic m6A methylation and expression patterns, particularly those involved in the sporulation phase. These genes tend to be upregulated when needed during sporulation and downregulated when not, with our data suggesting potential association between m6A patterns and expression changes across sporulation timepoints. The conjoint examination of m6A methylation and gene expression trajectories during yeast sporulation, together with functional annotation analysis, provides a comprehensive view of m6A’s regulatory roles in significant genes during sporulation phases. By studying selected significant genes, we gained deeper insights into the coordination between m6A patterns and gene function during sporulation. Ultimately, our study reveals that *de novo* genes exhibit attenuated yet parallel patterns of m6A compared to their established counterparts. This lower apparent m6A burden is largely explained by their shorter transcript length and sequence composition rather than by evolutionary age *per se*, suggesting that the structural features accompanying gene maturation, more than age alone, shape the epitranscriptomic profile of newly emerged genes.

## 2 Methods

### 2.1 *De novo* gene extraction

*De novo* gene identification is a complex and time-consuming process, often lacking standardized methods across studies. To focus on the core objectives of our research and avoid potential methodological discrepancies, we utilized a previously established set of *de novo* genes. We extracted *de novo* genes based on data from a previous study that characterized 84 recently originated *de novo* genes specific to *Saccharomyces cerevisiae* S288C, comprising 41 transcribed and 43 translated genes (Wu and Knudson, 2018). This study comprehensively compared different approaches to propose a reliable set of *de novo* genes. These genes, representing approximately 1% of the total genes in the Saccharomyces Genome Database (SGD) were obtained. This curated list of total 84 *de novo* genes was used for our entire comparative analysis of m6A modifications and sequence features.

### 2.2 Data acquisition and preprocessing

Raw sequencing data of m6A modification in yeast were obtained from a previously published m6A-seq2 study, which utilizes multiplexed m6A-immunoprecipitation of barcoded and pooled samples to increase throughput while reducing technical variability and provides sample-level relative quantifications (Dierks et al., 2021). The methodology employs immunoprecipitation (IP) samples to capture m6A-modified RNA sequences alongside input samples that serve as controls representing the total RNA population for normalization and background correction. We utilized m6A-seq2 datasets covering five continuous time points across meiosis from two biological replicates, plus a prophase-arrested positive control sample (*Ndt80*Δ/Δ). The time points span the full meiotic program: T1 (0 h, pre-meiotic), T3 (2 h, early prophase I), T5 (4 h, late prophase I), T7 (6 h, meiosis I division), and T9 (8 h, meiosis II/sporulation), with PC (*Ndt80*Δ/Δ, prophase arrest). The published dataset also includes a negative control (NC, *Ime4*Δ/Δ/*Ndt80*Δ/Δ), an m6A-null sample lacking the m6A methyltransferase *IME4*, which was excluded from downstream analyses as its substantially low IP and input read counts destabilize peak calling and differential m6A quantification.

FASTQ files of this paired-end data, containing replicates, were downloaded using the SRA Toolkit (Sayers et al., 2022). Quality assessment of raw reads was performed using FastQC v0.12.1 (Brown et al., 2017), and a comprehensive quality report was generated with MultiQC v1.14 (Ewels et al., 2016). Reads were cleaned using Fastp with default parameters. Post-cleaning quality was re-evaluated with FastQC and MultiQC to ensure high-quality data.

### 2.3 Reference genome and alignment

The *Saccharomyces cerevisiae* S288C genome (version R64-3-1) and its annotation file from SGD were used as the reference genome (Cherry et al., 1998). The diploid SK1 strain, which undergoes efficient and synchronous sporulation and meiosis (Chu et al., 1998), was used for the m6A-seq2 dataset (Dierks et al., 2021). S288C genome was selected over the SK1 reference in this study due to superior DNGs annotation coverage while producing consistent m6A results and high concordance between genome-based analyses, as shown in Supplementary Figure S1. This well-annotated genome ensures reliable mapping of sequencing reads. Alignment of cleaned reads was conducted using HISAT2 v2.2.1 with optimal parameters for yeast genome alignment (Kim et al., 2019). HISAT2 was chosen for its ability to handle spliced alignments efficiently. To assess sample correlation and alignment quality, DeepTools was used for visualization (Ramírez et al., 2014), and QualiMap for detailed quality metrics (García-Alcalde et al., 2012). Further alignment quality assurance was performed using Samtools (Li et al., 2009), and results were summarized with MultiQC.

### 2.4 Differential gene expression analysis

Gene expression levels were quantified from the m6A-seq2 input samples using StringTie v2.2.1 and featureCounts, which provided TPM (Transcripts Per Million) metrics across all samples (Pertea et al., 2016). Differential gene expression analysis was performed using the earliest meiosis stage (T1) as the reference point to calculate log2 fold change expression measurements for subsequent timepoints.

### 2.5 m6A peak calling and differential methylation analysis

Accurate identification of m6A peaks is essential for understanding their distribution and potential functional impacts. Here, m6A peaks were identified using the exomePeak2 R package (version 1.2.0). ExomePeak2 was chosen for its robust peak calling and differential methylation analysis capabilities (Meng et al., 2014). All replicate IP and input BAM files were provided as input to the main exomePeak2 function, allowing the software to model biological variability and technical differences across samples. It was configured to use full transcript information and ensure consistent peak calling across replicates. The score metric, defined as -log10 *p* was used for age group comparisons, as it reflects statistical confidence while minimizing sensitivity to gene length variations. For differential methylation analysis, the diff log2FC metric was applied, using T1 (0 h, the earliest meiotic timepoint) as the reference baseline, as it shares the same biological composition as later stages while representing the natural floor of meiotic m6A induction. In addition, the m6A Gene Index (m6A-GI) was calculated as the ratio of IP to input read counts across full gene bodies, with strand information preserved to ensure correct transcriptional orientation. For each gene, the sum of scores across all significant peaks was computed, assigning a value of zero to genes without detected peaks. Significant peaks for PCGs were extracted, and peaks specific to DNGs were identified using BEDTools intersect (Quinlan and Hall, 2010).

Following peak calling, we employed the R package ChIPseeker v1.34.1 to annotate peak locations, utilizing the Bioconductor yeast annotation library *TxDb.Scerevisiae.UCSC.sacCer3.sgdGene* as a reference. This gene model provides transcription start site (TSS) and transcription termination site (TTS) positions based on UCSC/SGD annotations. The *plotPeakProf()* function in ChIPseeker was used to analyze m6A peak distribution across gene bodies, automatically utilizing only genes with complete TSS and TTS annotations for metagene profile generation. Genes lacking complete coordinate information were implicitly excluded from the analysis by the software without requiring manual filtering (Yu et al., 2015). For genome wide normalized enrichment profiles IP and Input BAM files were converted to log2(IP/Input) ratio bigWig tracks using bamCompare from DeepTools (Ramírez et al., 2014). ComputeMatrix was then used to generate signal matrices across all gene bodies (±1 kb flanking regions) for each timepoint, and plotProfile was used to generate the resulting metagene profiles. For representative single gene tracks, bamCompare-derived log2(IP/Input) bigWig tracks were visualized directly across the gene body for multiple randomly selected genes.

### 2.6 Motif analysis

In yeast, the GAC motif is strongly associated with m6A methylation sites. To investigate its enrichment within m6A peak regions, motif analysis was performed using MEME-ChIP and Homer. Both tools consistently identified the GAC motif as a consensus sequence. *De novo* analysis using Homer further validated the significance of the GAC motif across all time points when comparing PCGs and DNGs. Motif visualization was generated using the R package ggseqlogo. To deepen the analysis, the k-mer technique was applied to quantify the occurrence of the GAC motif within RNA peak regions of both PCGs and DNGs, enabling unbiased identification of sequence-level patterns. Custom Python scripts were used to compute the median GAC motif density per 100 nt across different sporulation stages. A probabilistic approach quantified GAC enrichment by comparing its observed frequency in peaks to the expected frequency from overall nucleotide composition. To account for gene length differences between DNGs and PCGs, enrichment scores were normalized using: (GAC k-mer count/total sequence length)/(Freq(G) × Freq(A) × Freq(C)), correcting for length and background composition bias.

To further disentangle the determinants of m6A deposition, a multivariable ordinary least squares (OLS) regression was performed (statsmodels, Python) modeling the per-gene m6A score as a function of GAC motif density, log-transformed transcript length, log-transformed expression level, and evolutionary age as rank, with meiotic timepoint included as a categorical covariate. Standardized regression coefficients (β) were computed to enable direct comparison of effect sizes across predictors.

### 2.7 Functional enrichment analysis

Functional enrichment analysis provides biological context to gene sets, helping to interpret the potential roles and processes associated with m6A-modified genes. Functional enrichment analysis was conducted using the Database for Annotation, Visualization and Integrated Discovery (DAVID) to perform Gene Ontology (GO) analysis for all genes (Dennis et al., 2003). DAVID was selected for its comprehensive gene annotation database and robust statistical framework. This analysis provided a deeper functional understanding of the example genes discussed in the results section. We focused on biological process, molecular function, and cellular component categories to gain a comprehensive view of gene functions. Additional literature review was conducted to further interpret and contextualize the functional enrichment results, ensuring a thorough understanding of the biological implications of our findings.

### 2.8 Data visualization

Data visualizations were performed using Python and R programming languages. Additionally, we utilized SRplot, a free online platform for data visualization and graphing (Tang et al., 2023), and the CNSknowall platform (https://cnsknowall.com/#/HomePage), a comprehensive web service for biomedical data analysis and visualization. These tools enabled optimal representation and analysis of our research results.

## 3 Results

### 3.1 *De novo* genes exhibit reduced m6A modifications compared to other protein-coding genes

**FIGURE 1:**
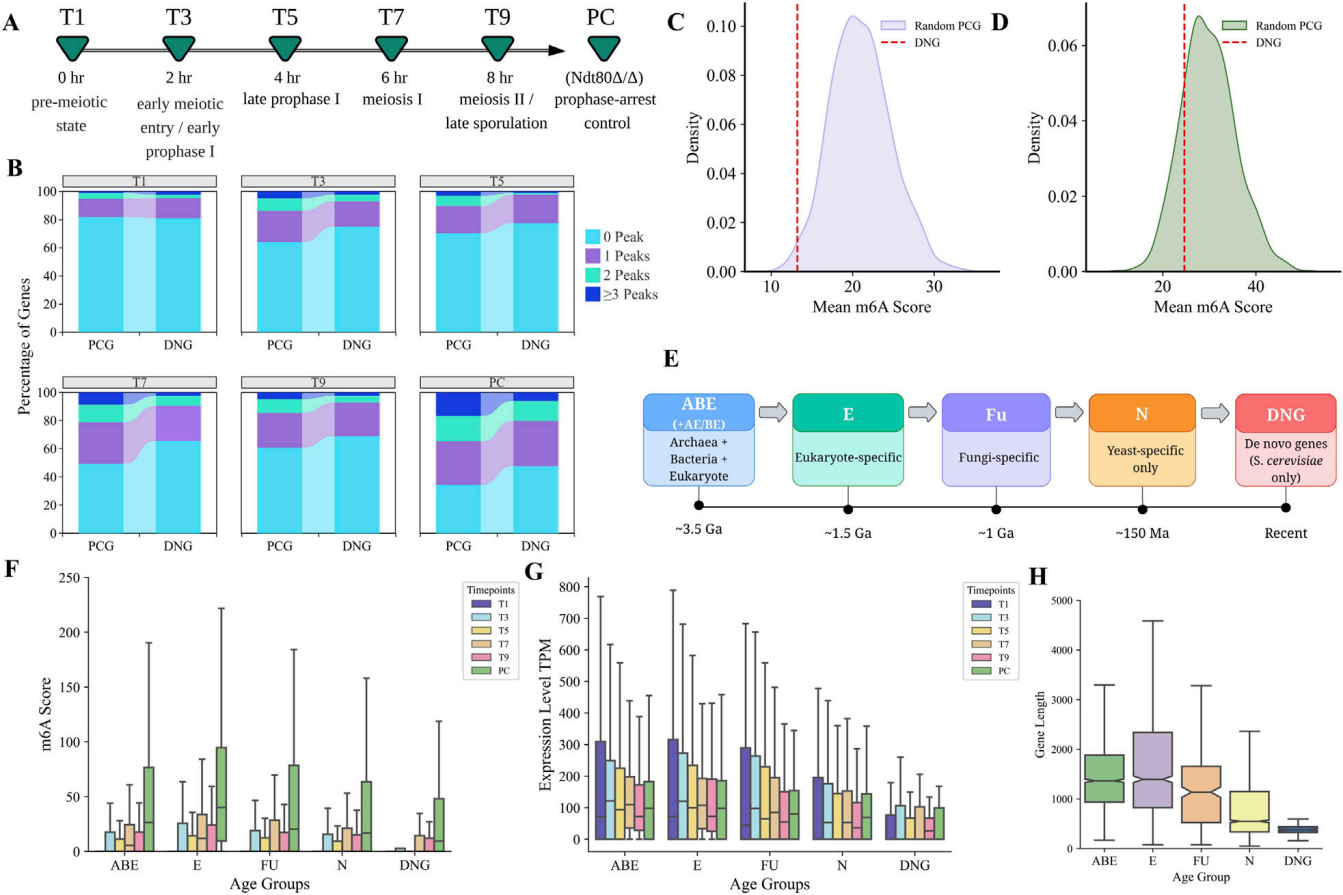
Comparative overview of m6A methylation patterns across PCGs, DNGs, and gene groups of varying evolutionary origins. **(A)** Schematic of the six sporulation samples: five continuous timepoints and a positive control sample, designated as T1 (0 h), T3 (2 h), T5 (4 h), T7 (6 h), T9 (8 h), and PC (Ndt80Δ/Δ). **(B)** Percentage of genes containing 0, 1, 2, or ≥3 m6A peaks across these timepoints in PCGs and DNGs. (C, D) Density plots representing the variability in m6A modification score across 1000 random subsets of PCGs, matched to DNGs by expression **(C)** and gene length **(D)** as controls; the red dashed line indicates the observed DNG mean. **(E)** Schematic of five evolutionary age groups arranged from oldest to youngest along an approximate evolutionary timescale: ABE (Archaea, Bacteria, and Eukaryotes; encompassing the ancestral AE and BE groups), E (Eukaryote-specific), Fu (Fungi-specific), N (yeast-specific), and DNG (de novo genes). **(F)** m6A levels as peak score across gene groups (ABE, E, FU, N, DNG) at various timepoints. **(G)** Expression level as TPM across these gene age groups. **(H)** Gene lengths of different gene age groups.

To explore the modification patterns and evolutionary aspects of m6A in newly emerged DNGs in yeast, we first sought to determine whether the RNAs of these genes are methylated differently from those of older, established PCGs. For this we utilized m6A-seq2 data samples (Dierks et al., 2021), covering five continuous individual time points (h) across meiosis from two biological replicates in addition to an Ndt80Δ/Δ (prophase arrested) positive control sample, referred to as T1, T3, T5, T7, T9 and PC respectively (Figure 1A; Supplementary Material S1). The total set of annotated protein-coding genes included 6,600 genes, from which 84 were labeled as DNGs (Wu and Knudson, 2018), resulting in 6,516 non-overlapping PCGs for our comparative analyses. The 84 DNGs selection was based on the criteria which require: (1) arising from non-coding genomic region, (2) absence of homologs in related species, (3) presence of syntenic non-coding orthologs, and (4) supporting evidence of expression or translation.

The m6A peak enumeration analysis across sporulation revealed distinct patterns between DNGs and PCGs (Figure 1B). Both gene sets showed temporal increases in m6A deposition over time, with the highest levels observed at PC, consistent with maximal m6A accumulation at prophase I arrest (Agarwala et al., 2012). However, DNGs consistently exhibited fewer peaks compared to PCGs. Specifically, in PCGs, approximately two-thirds of genes (∼4,460) harbored multiple peaks, while nearly one-third remained unmodified. In contrast, nearly half DNGs (∼39 genes) completely lacked m6A peaks, while the remaining half possessed one or more peaks. Cumulatively, PCGs averaged 4.02 peaks per transcription unit versus 2.62 for DNGs. These findings demonstrate that DNGs show a lower apparent per-gene m6A burden relative to PCGs, with clear differences in m6A peak abundance distribution (Supplementary Figure S2).

Noting the fact that DNGs are bioinformatically identified, mostly annotated as “dubious ORFs”, with few listed as “putative” or “uncharacterized” ORFs in the *Saccharomyces* Genome Database (SGD).We therefore determined whether translation status affects m6A levels within DNGs, by partitioning the DNG set into translated DNGs (n = 43, ribosome profiling RPKM ≥0.5) versus transcribed-only DNGs. However, no significant difference in m6A abundance was observed between these subsets (Supplementary Figure S3).

Given the challenges in accurately identifying DNGs and the potential for false positives in individual datasets, we analyzed an independent DNG dataset to validate our findings. This dataset contains 213 DNGs that show no homologs beyond *S. mikatae* (Blevins et al., 2021). We observed similar patterns of temporal increases in m6A deposition with consistently higher PCG methylation versus DNGs (Supplementary Figure S4). Stratification by translation status (transcribed, n = 116; translated, n = 97) showed no significant difference in m6A abundance within this DNG subset (Supplementary Figure S5).

To compare the proportion of significant m6A modification sites between the two gene sets while accounting for potential confounders, we used a permutation approach. For each test, matched PCG subsets were randomly sampled to generate a null distribution of mean m6A scores, against which the observed mean m6A score of the DNG set was compared (depicted as a vertical line). First, controlling for gene expression, expression-matched sampling yielded a significant difference (two-tailed permutation test, n = 1,000; *p* = 0.02; Figure 1C), indicating that the reduced methylation in DNGs is not attributable to expression-level differences alone. DNGs are markedly shorter than PCGs (mean length: 422 bp vs. 1,492 bp); accordingly, length-matched comparison revealed no significant difference in m6A scores between the two groups (two-tailed permutation test, n = 1,000; *p* = 0.35; Figure 1D), demonstrating that transcript length accounts for a substantial proportion of the apparent m6A deficit observed in DNGs.

Given that gene length covaries with evolutionary age, we next examined whether m6A deposition follows a broader evolutionary gradient. Yeast genes were stratified into five phylogenetic age groups (Figure 1E). The oldest genes, shared across Archaea, Bacteria, and Eukaryotes (ABE), represent the most ancient group. The subsequent groups include Eukaryote-specific genes (E), Fungi-specific genes (Fu), and genes specific to yeast (N) (Kim and Marcotte, 2008). The youngest group comprises DNGs that have emerged recently in *S. cerevisiae.* A weak but statistically significant negative association between evolutionary age and m6A score was detected across all meiotic time points (two-sided Spearman’s ρ = −0.030 to −0.074, all *p* < 0.05; Figure 1F), with ABE genes displaying the highest scores and DNGs the lowest. These differences were significant across all age groups (Kruskal–Wallis, *p* < 0.001; pairwise comparisons, Bonferroni-corrected *p* < 0.05). RNA expression levels exhibited a stronger declining trend with decreasing evolutionary age across all time points (two-sided Spearman’s ρ = −0.150 to −0.169, all *p* < 10^−34^; Figure 1G), with significant differences across all pairwise group comparisons (all *p* < 10^−38^).

Since older genes are also longer (Figure 1H), partial correlation analyses were performed to isolate the contribution of evolutionary age to m6A scores independent of transcript length. Evolutionary age remained weakly but significantly associated with m6A scores after controlling for gene length at T1–T9 (two-sided partial ρ = −0.063 to 0.057, all *p* < 0.001), though this association was not significant at the PC timepoint (*p* = 0.52).

To assess the robustness of these findings, the analysis was repeated using an independent age classification system that incorporates both evolutionary origin and gene duplication timing (Doughty et al., 2020). This alternative stratification reproduced the weak but significant declining trends in m6A score (two-sided Spearman’s ρ = −0.035 to −0.107, all *p* < 0.01) and expression level (two-sided Spearman’s ρ = −0.041 to −0.064, all *p* < 0.01) across evolutionary age (Supplementary Figure S6). Partial correlation analysis with this classification confirmed that the weak association between evolutionary age and m6A score persisted across all meiotic time points after controlling for gene length (two-sided partial ρ = −0.092 to −0.137, all *p* < 10^−12^). Taken together, these results indicate that the lower apparent per gene m6A burden of DNGs is substantially influenced by transcript length, while evolutionary age shows only a weak residual association with m6A score after accounting for length. This modest age-related contribution is not consistently observed across all timepoints and classifications, and we therefore interpret it cautiously as a possible contributory rather than determinative factor in the m6A profile of newly emerged genes. Gene identifiers for these two age classification categories with associated methylation, expression, and length metrics are listed in Supplementary Material S2.

### 3.2 *De novo* genes follow the canonical trend of m6A density patterns along transcripts with distinct gene body enrichment

**FIGURE 2:**
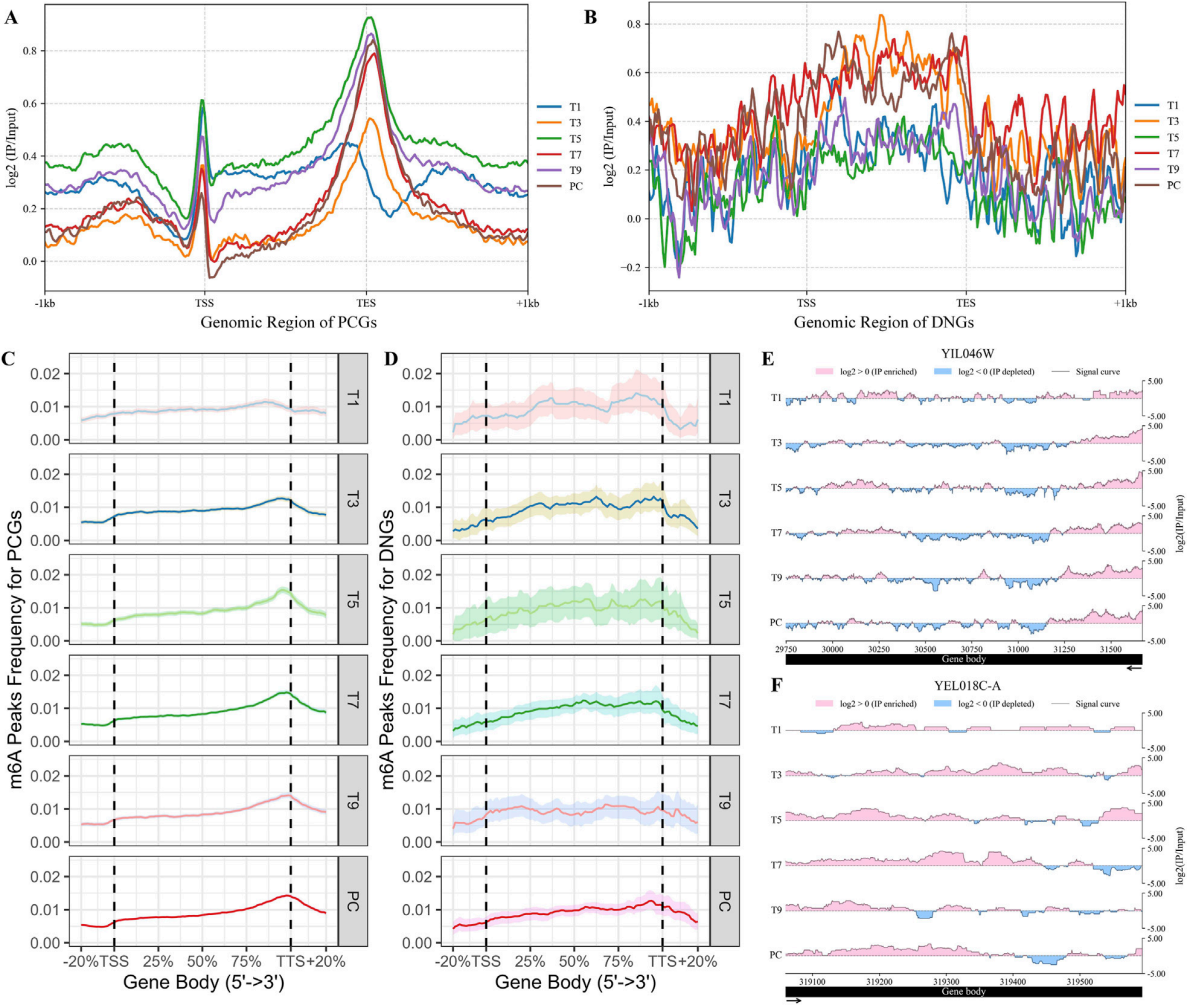
Distribution of m6A across RNA transcripts during sporulation. **(A,B)** Genome wide log2(IP/Input) metagene profiles for PCGs and DNGs across all timepoints (T1-PC); X-axis represents relative genomic position spanning ±1 kb around the gene body from transcription start site (TSS) to the transcription termination site (TTS). **(C,D)** Peak frequency of m6A across standardized transcript length (TSS−20% to TTS+20%) for PCGs and DNGs over the gene body (5′to 3′); dashed lines mark TSS and TTS. **(E,F)** Representative single gene log2(IP/Input) tracks for YIL046W (PCG) and YEL018C-A (DNG); X-axis shows genomic coordinates (bp) of gene architecture, strand direction indicated by arrow below the gene body. Y-axis limits are consistent within each panel group.

Since m6A methylation exhibits characteristic positional preferences that influence RNA fate, we investigated whether DNGs display similar spatial distribution patterns as established genes. To address potential confounds from 3′coverage bias and uneven fragmentation, we generated genome wide, input normalized log2(IP/Input) metagene profiles (Figure 2A; PCGs, Figure 2B; DNGs). The normalized enrichment profiles show that m6A signal in PCGs progressively increases from the TSS, peaking sharply within the final ∼20% of the transcript, with maximum enrichment at the TTS. DNGs, by contrast, lack a comparable defined TTS peak, instead showing a broad increase in signal across the gene body without sharp terminal enrichment. In the peak count frequency analysis of called m6A peaks (Figures 2C,D), PCG peak density follows a similar trend from the TSS, peaking at the TTS, and is slightly more pronounced during later sporulation stages, particularly PC, while DNGs retained TTS-proximal enrichment but with a more dispersed, variable distribution. The prominence of these signals during late meiotic stages likely reflects m6A’s role in transcriptome remodeling, marking mRNAs for decay or translational repression to facilitate efficient progression through sporulation (Yang et al., 2020).

To test whether this dispersed DNG profile reflects a distinct biological property or simply DNGs’ short length and low expression, we generated length and expression matched PCG controls (n = 72; nearest neighbour matching within a ±20% length window, then expression matched within that pool; matched PCG median length 372bp vs. DNG median 378bp), and stratified all PCGs into four length quartiles (Supplementary Figure S7). Matched PCGs and the shortest quartile (Q1, 51 to 567bp, spanning the DNG length range) both display flat profiles similar to DNGs, while longer quartiles (Q3, Q4) show progressively sharper TTS enrichment. This indicates the dispersed DNG distribution is partly attributable to short transcript length, a confound shared with short PCGs, rather than an exclusive property of gene age.

Beyond the length confound, 3′-end enrichment in yeast is well established, with m6A sites concentrated near stop codons and 3′regions (Meyer et al., 2012; Schwartz et al., 2013). In yeast, 3′m6A methylation can reduce mRNA levels during meiosis, promoting transcript turnover (Bushkin et al., 2019). Due to compact gene architecture with limited introns and short or poorly annotated UTRs, m6A deposition in yeast largely occurs within coding sequences, in contrast to eukaryotes with extended 3′UTRs where it more strongly regulates stability and translation. *De novo* proteins are generally smaller than duplicated proteins (Montañés et al., 2023), and young genes tend to be highly disordered with exaggerated gene-like structural properties (Wilson et al., 2017). The variable m6A distribution observed in DNGs may therefore reflect their less optimized nature and shorter transcript lengths, characteristics of recently evolved genes.

Representative single gene tracks (Figures 2E,F) of YIL046W (PCG) shows 3′end enrichment, while YEL018C-A (DNG) shows dispersed signal across the gene body region. Multiple examples (3 PCGs, 3 DNGs; Supplementary Figure S8) show individual genes can display heterogeneous topology despite consistent population trends, consistent with reported m6A variability (Bodi et al., 2015).

### 3.3 Consensus GAC methylation motif of yeast is less pronounced in *de novo* genes

**FIGURE 3:**
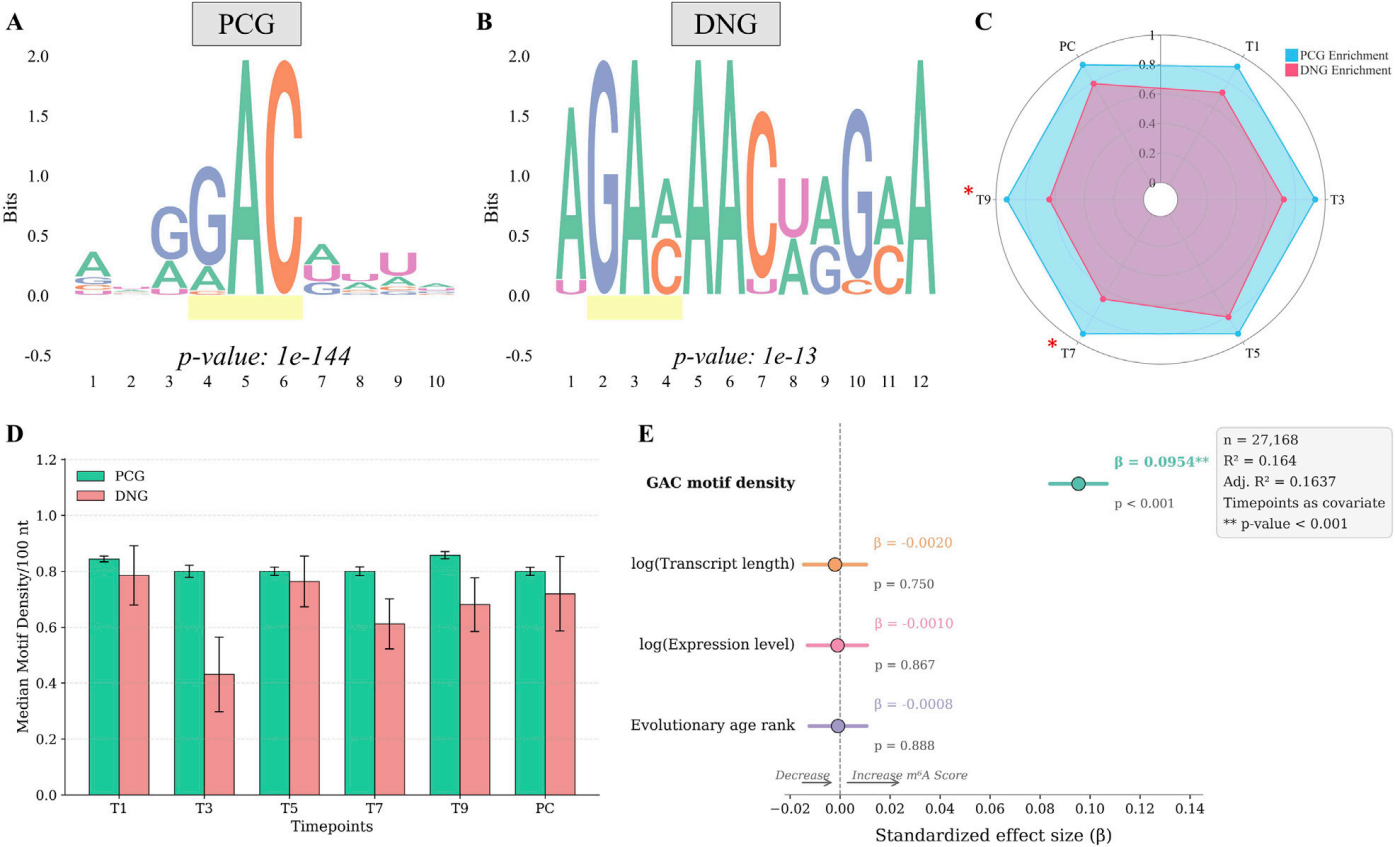
Consensus GAC motif analysis and comparison between DNGs and PCGs. **(A,B)** The GAC consensus motif associated with m6A RNA modification peaks (highlighted in yellow) within the identified *de novo* motifs of PCGs and DNGs, respectively. **(C)** The enrichment levels of GAC motifs (k-mer) occurrence in DNG peaks compared to that in PCG peaks. **(D)** Median GAC motif density per 100 nucleotides (nt) in DNG and PCG groups. Error bars represent standard error of the mean (SEM). **(E)** Multivariable OLS regression model (n = 27,168; R^2^ = 0.164) showing standardized effect sizes (β) for GAC motif density, log (transcript length), log (expression level), and evolutionary age rank as predictors of m6A score, with meiotic timepoint included as a covariate.

m6A modifications in RNA sequences are typically associated with specific consensus motifs, which vary across species and play a crucial role in regulating m6A function. To explore the implications of these motifs in yeast DNGs, we analyzed the sequences of methylated peaks from both PCGs and DNGs across all individual sporulation time points. Figures 3A,B illustrate the top-enriched motif in PCGs and DNGs, respectively, from *de novo* motif discovery. Notably, the GAC motif was significantly enriched in both PCGs (*p* = 1.0 × 10^−144^) and DNGs (*p* = 1.0 × 10^−13^). This qualitative examination of the motif logos reveals that PCGs display a well-defined GAC consensus sequence with strong positional conservation, while DNGs exhibit a comparatively more degenerate motif pattern with lower information content scores, indicating less stringent sequence requirements for m6A deposition.

To further explore the GAC motif’s occurrence in the two gene sets, we performed a k-mer analysis to quantify its enrichment and density within m6A peak sequences at various time points. The enrichment analysis of GAC motifs was performed using length-normalized metrics to eliminate potential confounding effects from the typically shorter sequences of DNGs compared to PCGs. The analysis revealed a notably higher enrichment of the GAC motif in PCGs compared to DNGs, most significant at later meiotic stages (Figure 3C, *p* = 0.00012, two-sided Mann–Whitney U test). This difference was also significant when comparing the median GAC motif density per 100 nt between the two gene sets (Figure 3D, *p* = 5.23 × 10^−4^, two-way ANOVA). PCGs exhibited consistently higher motif density (0.80–0.87/100 nt) compared to DNGs (0.43–0.79/100 nt) across all individual timepoints. This quantitative disparity suggests that while the GAC motif facilitates m6A modification in both gene classes, it plays a more prominent regulatory role in established PCGs than in evolutionarily younger DNGs.

To further disentangle the contributions of sequence composition, transcript length, expression level, and evolutionary age to m6A deposition, we performed a multivariable ordinary least squares (OLS) regression analysis across all peaks (n = 27,168; R^2^ = 0.164), with meiotic timepoint included as a covariate (Figure 3E). GAC motif density emerged as the only significant predictor of m6A score (β = 0.0954, *p* < 0.001), while transcript length (β = −0.0020, *p* = 0.750), expression level (β = −0.0010, *p* = 0.867), and evolutionary age rank (β = −0.0008, *p* = 0.888) showed no significant independent associations. These results indicate that sequence composition, specifically GAC motif density, rather than evolutionary age or transcript features, is the primary determinant of m6A deposition patterns across sporulation. The lower GAC density and more degenerate motif pattern in DNGs may reflect their potential reliance on transcript-level features such as RNA secondary structure accessibility rather than strict sequence determinants; however, as RNA structure was not directly assessed here, this remains a hypothesis warranting future experimental validation. Together, these findings indicate that the comparatively sparse and less canonical m6A signatures of DNGs are shaped chiefly by their sequence composition, consistent with the established influence of m6A on gene expression, mRNA stability, and cellular function (Fu et al., 2014).

### 3.4 m6A levels of *de novo* genes follow a parallel trend during sporulation

**FIGURE 4:**
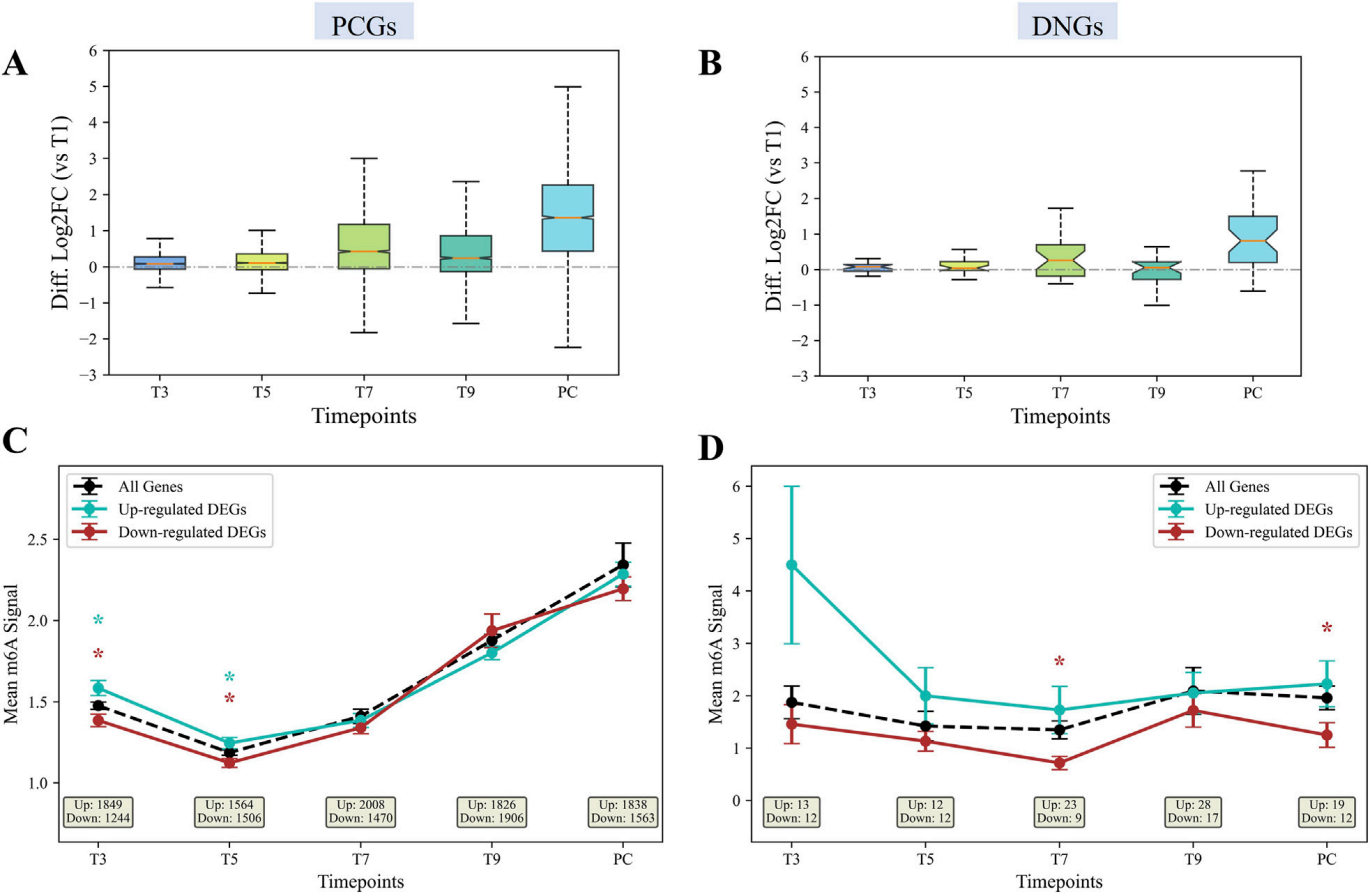
Dynamic m6A modifications during sporulation timepoints. **(A,B)** Temporal changes in m6A modifications shown as differential Log2FC for each timepoint (T3, T5, T7, T9, PC) relative to the earliest timepoint (T1) for PCGs and DNGs, respectively. Error bars represent the minimum and maximum values, excluding outliers, for each timepoint. **(C,D)** Mean m6A signal as m6A gene index for DEGs categorized as upregulated or downregulated, compared to the average signal across all genes for PCGs and DNGs, respectively. Significant differences (*p* < 0.05), assessed using the Mann–Whitney U test, are indicated by blue asterisks for upregulated DEGs and red asterisks for downregulated DEGs.

In yeast, m6A modifications are deposited primarily in prophase, accompanied by maximal MTC activity (Ensinck et al., 2023). Such a trend is captured by our cross-stage analysis (Figures 4A,B). m6A methylation patterns were analyzed across meiotic timepoints (T3, T5, T7, T9, PC) relative to the earliest timepoint (T1) for PCGs and DNGs. Both gene classes exhibited progressive m6A accumulation throughout meiotic progression, with maximum levels at prophase (PC). PCGs showed substantial m6A enrichment peaking at PC (median differential Log2FC ∼1.5), while DNGs displayed more modest but detectable increases (median differential Log2FC ∼0.8). The peak m6A levels at PC correspond to maximal expression and activity of MTC components, particularly Ime4, which drives extensive methylation during this critical meiotic stage. Conversely, the minimal m6A levels at T3 likely reflect decreased MTC component expression and increased activity of reader proteins such as Pho92, which facilitate targeted mRNA degradation (Varier et al., 2022). This progressive trend of m6A levels, in both PCGs and DNGs is also captured by the differential Log2FC values between adjacent timepoints (Supplementary Figure S9).

Differentially expressed genes (DEGs) during sporulation may contribute to the regulation of meiosis in yeast. To explore whether these genes exhibit exceptional m6A modifications during meiosis, we quantified methylation enrichment across sporulation timepoints for upregulated and downregulated PCGs and DNGs using the m6A gene index, calculated as the ratio of IP to input reads per gene. This metric normalizes for expression differences, allowing unbiased comparison across genes with varying transcriptional levels. Figures 4C,D illustrate that upregulated genes generally display higher m6A signals than both downregulated genes and the genomic baseline, with the most pronounced enrichment observed during early timepoints. This tendency is particularly evident in PCGs, where several timepoints show statistically significant differences (two-sided Mann–Whitney U test, *p* < 0.05; blue asterisks), suggesting that elevated m6A levels may be associated with the regulation of gene expression during meiosis. In contrast, DNGs exhibit weaker but consistent differences in m6A enrichment between upregulated and downregulated DEGs, likely correlative with their lower expression levels and nascent regulatory architecture. Notably, while both PCGs and DNGs exhibit peak m6A accumulation at prophase (PC) (Figures 4A,B), the temporal patterns of significant m6A differences between upregulated and downregulated DEGs differ. PCGs show early significance (T3, T5) (Figure 4C) reflecting molecular “pre-patterning” where m6A readers are recruited co-transcriptionally to mark transcripts for subsequent regulation (Meyer et al., 2012). In contrast, DNGs display later significance (T7, PC) (Figure 4D), potentially indicating their reliance on more direct, contemporaneous m6A-mediated regulation coinciding with peak MTC activity.

These results are consistent with m6A acting as a post-transcriptional modulatory layer that fine-tunes gene expression, influences transcript stability and turnover rather than serving as a primary driver of expression changes (Fu et al., 2014; Roignant and Soller, 2017; Heck and Wilusz, 2019; Xie et al., 2025; Yang et al., 2025).

### 3.5 Key *de novo* genes identified through conjoint m6A and gene expression analysis in sporulation

To further understand the functional relationship between m6A methylation and gene expression during sporulation, we analyzed both m6A-seq and RNA-seq datasets to identify differentially methylated genes (DMGs) and differentially expressed genes (DEGs) across all timepoints in reference with the earliest timepoint (T1). Using a quadrant analysis approach, genes were classified based on their differential methylation (m6A) and differential expression (RNA) profiles. A ±0.5 log2 fold change threshold, together with a *p*-adjusted <0.05 cutoff, was applied to both m6A and expression data, and genes were assigned to four regulatory categories: Hyper-Up (increased methylation and expression), Hyper-Down (increased methylation, decreased expression), Hypo-Up (decreased methylation, increased expression), and Hypo-Down (decreased methylation and expression).

**FIGURE 5:**
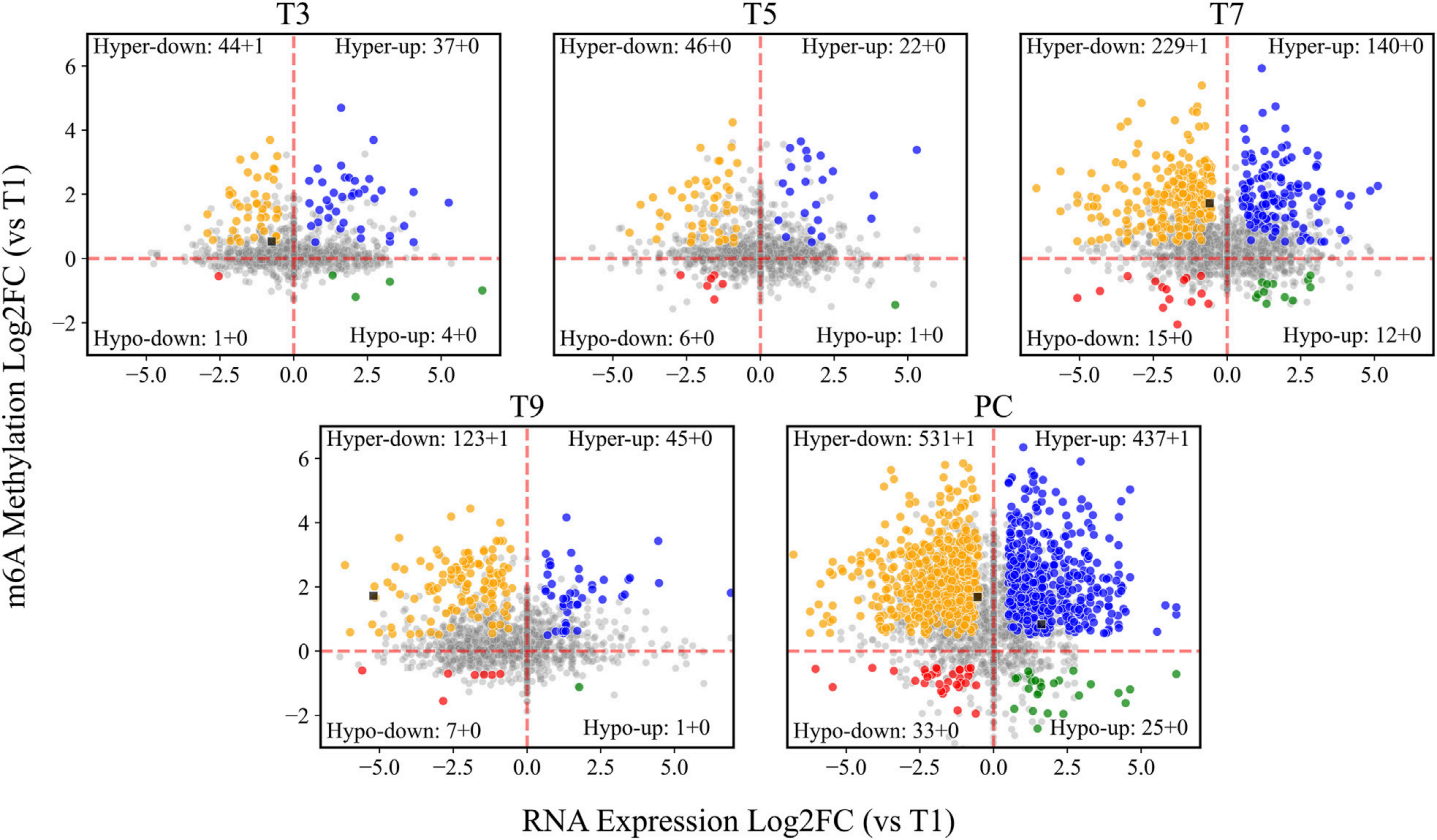
Quadrant analysis of differential RNA expression and m6A methylation during sporulation timepoints, depicting the distribution of all genes (represented as dots) across the expression and m6A methylation axes. The quadrants are defined as Hyper Down (yellow), Hyper Up (blue), Hypo Down (red), and Hypo Up (green). *De novo* genes (DNGs) within these quadrants are shown in black, while gray dots represent genes with non-significant differential expression or m6A methylation.

The significant gene clusters show a progressive increase across consecutive timepoints relative to T1, reflecting the context dependence of each quadrant and collectively supporting a modulatory role for m6A during sporulation (Figure 5). Hyper-Down genes, the dominant quadrant, indicate that m6A accumulation is systematically associated with transcript clearance rather than induction, a pattern consistent with m6A-driven mRNA decay mediated by the yeast reader Pho92 via the CCR4-NOT complex (Varier et al., 2022). The Hyper-Up population supports the notion that m6A deposition occurs co-transcriptionally, such that highly transcribed mRNAs proportionally accumulate more m6A marks, which in turn modulates their translation efficiency (Akhtar et al., 2021). The Hyper-Down and Hyper-Up populations of genes demonstrate that m6A does not uniformly drive expression changes. Rather, its functional consequences are context-dependent, determined by the identity of downstream reader proteins rather than the modification itself (Hong et al., 2022).

Three unique DNGs meeting the threshold criteria (highlighted as black squares) were identified, two exhibiting unknown functional annotations while one was associated with cellular membrane components and transmembrane structures. Although restricted in number, the identification of these candidate m6A-regulated DNGs is consistent with the possibility that evolutionarily nascent genes can be subject to epitranscriptomic regulation during developmental transitions. This exploratory observation aligns with emerging paradigms wherein regulatory integration, rather than expression magnitude, may contribute to functional incorporation of young genes into biological networks (Cai and Petrov, 2010; Chen et al., 2013). This pattern is also consistent with the established function of m6A modification as a key post-transcriptional regulatory layer that can influence mRNA stability and translation efficiency during developmental transitions like meiosis and sporulation (Meyer and Jaffrey, 2014), although confirming this role in DNGs would require targeted functional validation.

**FIGURE 6:**
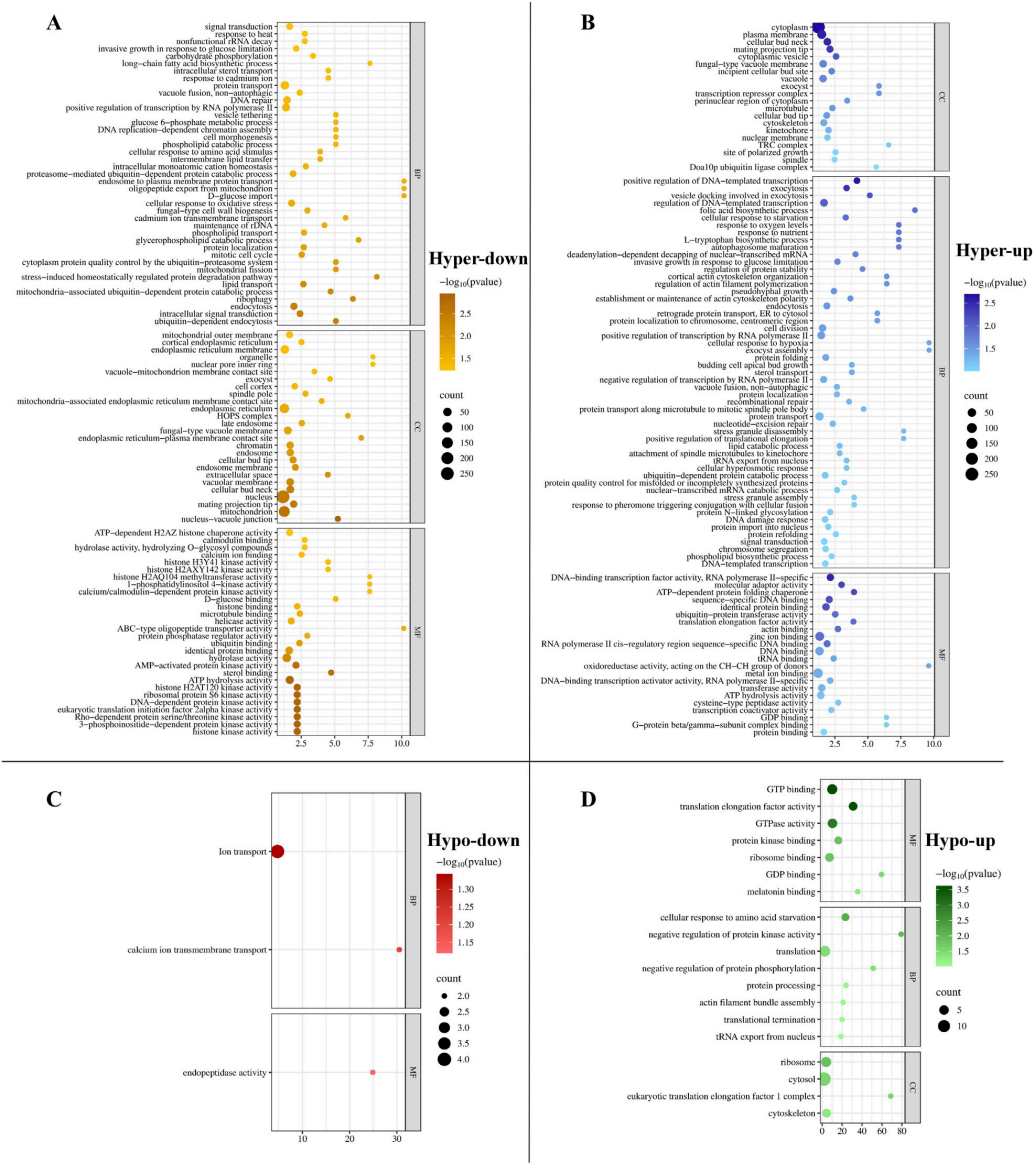
GO Enrichment analysis of significant genes across quadrants. **(A–D)** Dot plots showing GO terms enriched in Hyper-Down **(A)**, Hyper-Up **(B)**, Hypo-Down **(C)**, and Hypo-Up **(D)** genes. For all panels: x-axis represents gene count, y-axis lists enriched GO terms categorized by biological process (BP), molecular function (MF), and cellular component (CC), dot color indicates -log_10_ (*p*) for statistical significance; dot size is proportional to gene count.

To gain insights into the biological significance of the methylation-expression profiles of PCGs identified through quadrant analysis, we performed functional enrichment analysis using Gene Ontology (GO) terms. This analysis was conducted for all four gene categories, encompassing all timepoints, and the findings are displayed in Figure 6.

Hyper-Down genes were enriched in metabolic processes including long-chain fatty acid biosynthesis, carbohydrate phosphorylation, and glucose-responsive pathways, alongside oxidative stress response and ubiquitin-dependent proteolysis. The suppression of these transcripts under elevated m6A is consistent with m6A-driven mRNA potentially decay acting as a mechanism to actively remodel the metabolic landscape as cells commit to sporulation, redirecting resources away from vegetative functions. Hyper-Up genes were enriched in transcription regulation, DNA-templated processes, actin cytoskeleton organization, and protein folding, indicating that co-transcriptional m6A accumulation on highly expressed mRNAs does not impair but accompanies the upregulation of core cellular machinery required to sustain meiotic progression. Hypo-Down genes were enriched in ion transport, suggesting that m6A loss accompanies the coordinated silencing of ion homeostasis pathways dispensable during sporulation. Hypo-Up genes were enriched in translation, protein kinase activity, and cellular response to amino acid starvation, indicating that subsets of protein production and stress-adaptive genes are activated independently of m6A gain. The functional identity of each quadrant thus reflects discrete and non-overlapping biological programs, consistent with the context-dependent nature of m6A regulation observed throughout sporulation.

### 3.6 Differential m6A methylation and gene expression trajectories during yeast sporulation

Our analysis of m6A methylation and gene expression across multiple yeast sporulation timepoints reveals distinct trajectories between PCGs and DNGs, highlighting both pervasive and gene-specific regulation patterns during meiosis. The average differential change (log2FC) in m6A modification and expression shows variability, reflecting positive and negative regulation across the different phases of sporulation, with genes displaying unique regulatory trends based on their biological roles.

**FIGURE 7:**
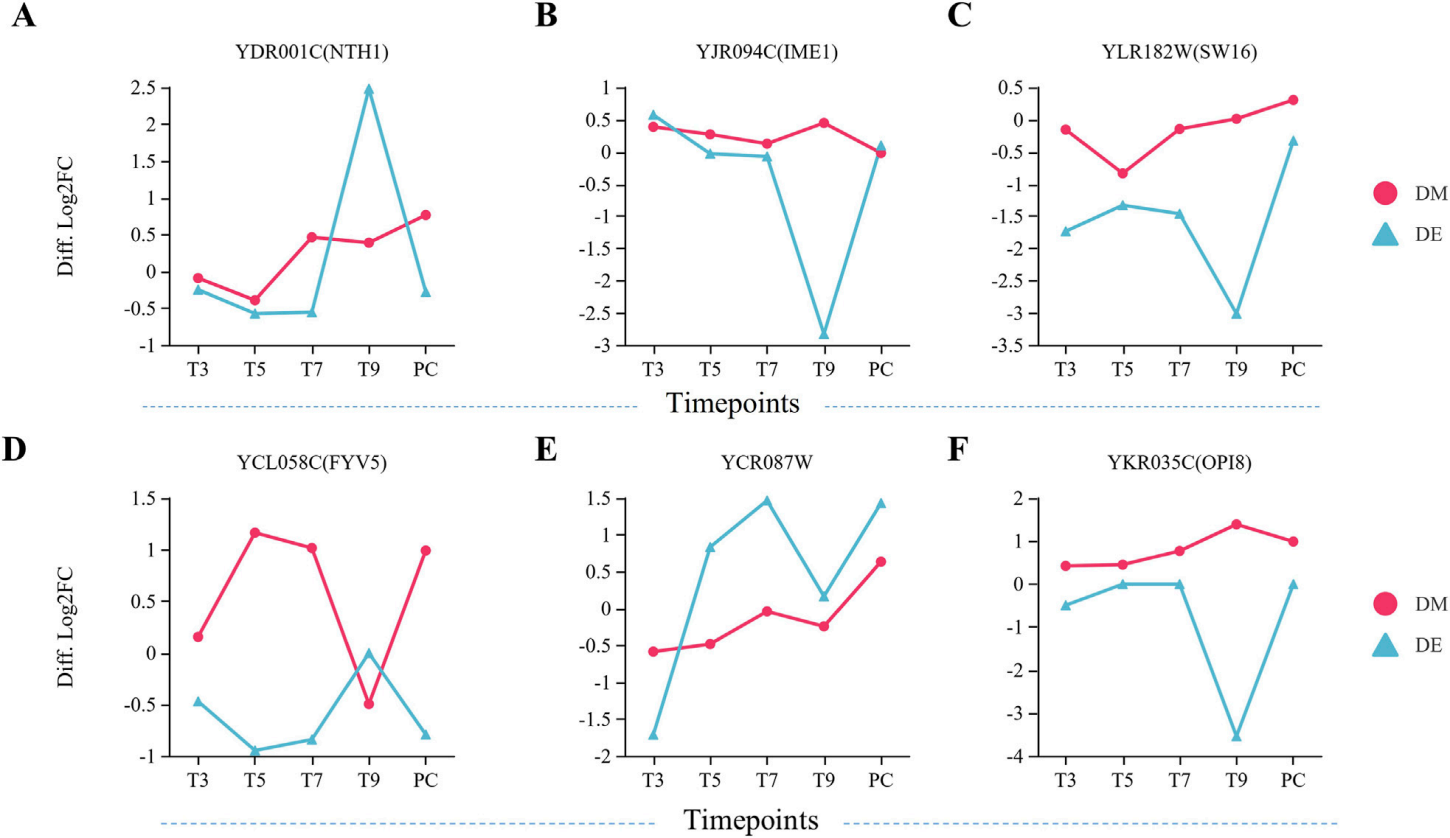
Differential m6A methylation (DM) and differential expression (DE) across sporulation timepoints. **(A–C)** Temporal DM and DE trends for representative PCGs, including *YDR001C* (*NTH1*), *YJR094C* (*IME1*), and *YLR182W* (*SW16*). **(D–F)** Temporal DM and DE trends for representative DNGs, including *YCL058C* (*FYV5*), *YCR087W*, and *YKR035C* (*OP18*). All values were normalized using Z-score transformation to allow comparison of relative changes over time.

In PCGs (Figures 7A–C), representative genes selected from the quadrant analysis show distinct methylation-expression dynamics. *YDR001C* (*NTH1*) exhibits an early DM increase preceding a peak in expression at T9, suggesting that m6A accumulation precedes and may prime post-transcriptional regulation of this transcript during mid-sporulation. *NTH1* encodes a neutral trehalase involved in trehalose metabolism, critical for spore viability and stress resistance (Nwaka et al., 1995). *YJR094C* (*IME1*) shows an early decline in both DE and DM from T3 onward, with both remaining suppressed through later timepoints. IME1 is a master transcriptional activator of early meiotic genes, and this pattern is consistent with its known role as an early meiotic factor whose transcript is rapidly cleared following initiation of the meiotic cascade, with m6A potentially contributing to its timely degradation (Park Z. et al., 2023). *YLR182W* (*SWI6*) shows progressive increases in both methylation and expression peaked at PC, which can be related to the fact that *SWI6* encoding a transcription cofactor essential for G1/S transition and meiotic recombination gene expression (Nakayama et al., 2000).

In DNGs (Figures 7D–F), more variable expression and m6A patterns emerge, consistent with their evolutionary novelty. *YCL058C* (*FYV5*) exhibits coordinated upregulation of methylation and expression during early-mid sporulation (T5-T7), followed by inverse regulation at later stages. *FYV5* mediates crosstalk between reproduction and vegetative growth through interaction with Snf1p, and its dynamic m6A regulation likely supports the metabolic transition from vegetative growth to sporulation (Clancy et al., 2002). *YCR087W* shows sustained expression upregulation with fluctuating m6A levels, suggesting complex post-transcriptional regulation throughout sporulation. *YKR035C* (*OPI8*) demonstrates progressive methylation increases alongside stable expression until T9, where expression drops dramatically while methylation remains elevated. This pattern is suggestive of m6A-mediated transcript destabilization or translational repression during late sporulation phases.

Therefore, while methylation and expression trajectories show gene-specific patterns, the regulation appears to be tailored to each gene’s biological function, with m6A modifications contributing to the coordinated expression dynamics of both PCGs and DNGs during sporulation.

## 4 Discussion

*De novo* genes (DNGs), representing newly emerged genetic elements, serve as fascinating windows into the evolutionary processes of genetic innovation (Schlötterer, 2015). These genes, which arise from previously non-coding sequences, provide unique opportunities to study the acquisition of regulatory features during gene evolution. Among various regulatory mechanisms, RNA modifications, particularly N6-methyladenosine (m6A), have emerged as crucial regulators of gene transcription and mRNA metabolism across organisms (Liu et al., 2019; Hou et al., 2022). Unlike epigenetic DNA and histone modifications that primarily influence transcriptional events, reversible RNA methylation impacts post-transcriptional regulation, directly affecting protein production and mRNA fate (Khan et al., 2013; Fu et al., 2014). Our study provides a comprehensive analysis of m6A modification patterns in DNGs compared to established PCGs during yeast sporulation. Since m6A modifications are exclusively deposited during meiosis in this model organism, our findings offer novel insights into the evolutionary dynamics of RNA modifications and their regulatory roles in gene transcription. Our investigation provides a unique perspective on the m6A profiles of newly emerged DNGs in comparison to their established counterparts, extending the exploration of evolutionary patterns from histone modifications to mRNA modifications.

Our findings suggest that the lower apparent m6A burden observed in DNGs is primarily attributable to their shorter transcript length and the resulting reduced sensitivity of peak detection, rather than to an inherent difference in evolutionary regulatory complexity. Although DNGs exhibited fewer m6A peaks per gene and approximately half lacked detectable m6A peaks, these differences were no longer significant after controlling for transcript length (Figure 1D). Furthermore, multivariable analysis identified GAC motif density, rather than evolutionary age, as the strongest predictor of m6A deposition (Figure 3E). These observations are broadly consistent with previous studies proposing that regulatory features, including post-transcriptional modifications, accumulate over evolutionary time (McLysaght and Hurst, 2016). However, our results indicate that this relationship is largely mediated by structural and sequence characteristics associated with gene age, particularly transcript length and motif composition, rather than by evolutionary age itself acting as an independent determinant of m6A methylation. Notably, this gene-level approach to m6A analysis is of paramount importance. As gene-level m6A modifications likely carry greater functional significance than site-specific alterations. A corroborating study in yeast have shown that while only 5%–8% of individual m6A sites are subject to purifying selection, a substantially higher proportion (10%–19%) of genes bearing m6A modifications are conserved at the gene level (Liu and Zhang, 2018). These findings underscore the evolutionary importance of m6A modifications in maintaining gene function and regulation. Both DNGs and PCGs showed enrichment of m6A peaks at the 3′end, highlighting the critical role of 3′UTR m6A modifications in mRNA stability, localization, and translation efficiency (Meyer et al., 2012). However, DNGs also showed dispersed m6A enrichment in CDS, that may stem from their structural and sequencing simplicity, which sets them apart from mature genes. Such localization is largely influenced by the exon junction complex, specifically the EIF4A3 component, which has been shown to block METTL3-mediated m6A modifications near exon-exon junctions within CDS, preventing m6A deposition in regions close to splice junctions and shaping the m6A topology along the transcript (Yang et al., 2022). The lower peak density in DNGs likewise tracks with their reduced GAC motif content and simpler sequence composition, which provide fewer of the determinants required for efficient methyltransferase recognition, rather than indicating an intrinsic regulatory deficit tied to gene age. The observed increasing 3′UTR enrichment during meiosis aligns with the role of m6A in regulating mRNA decay and translation during dynamic cellular processes, suggesting a fine-tuned mechanism for controlling gene expression during critical stages of sporulation (Agarwala et al., 2012).

Our motif analysis confirmed the presence of the GAC motif in both DNGs and PCGs, consistent with previous yeast studies (Schwartz et al., 2013). However, the median GAC motif density per 100 nt differed significantly between the two gene sets (Figure 3D), and multivariable regression identified this density as the principal determinant of m6A score, exceeding the contributions of transcript length, expression level, and evolutionary age (Figure 3E). Differences in motif enrichment and proportions within m6A peak sequences between the two gene sets indicate that DNGs possess fewer of the canonical sequence determinants required for efficient m6A deposition. Our results suggest that DNGs, with relatively relaxed selective constraints, exhibit a lower frequency of canonical GAC motifs compared to genes under higher selective pressure. These findings highlight the role of m6A modification sites as key regulatory elements, conserved through evolutionary selection, especially in genes essential for cellular function (Neme and Tautz, 2013; Zhang et al., 2020).

The dynamics of m6A levels during sporulation revealed intriguing patterns. The surge in m6A levels during meiosis with highest at PC (the prophase arrested stage) aligns with the formation of the MTC early in meiosis. This complex, comprising components such as Ime4, Mum2, Vir1, Kar4, and Slz1, stabilizes during meiotic prophase and is responsible for the rise in m6A levels (Ensinck et al., 2023). The declining m6A levels in earlier timepoints can be attributed to the involvement of m6A reader proteins, such as Pho92, which binds to specific mRNAs in an m6A-dependent manner during meiotic prophase, promoting downregulation of these transcripts (Schwartz et al., 2013; Scutenaire et al., 2023). Lower m6A levels in DNGs are likely driven by their shorter transcript length and reduced GAC motif density, which together limit opportunities for m6A deposition. In contrast, pseudogenes often exhibit higher m6A levels, which may promote transcript degradation and reduce interference with cellular regulatory networks. Consistent with this, m6A-mediated cytosolic decay has been proposed as a mechanism for clearance of nonfunctional RNAs (Fitzsimmons and Batista, 2019). These findings suggest that extensive m6A-mediated regulation may be progressively acquired during gene evolution. However, since transcript length and sequence composition strongly influence m6A deposition, the extent to which this pattern reflects evolutionary age remains uncertain. Further studies are needed to clarify how newly emerged genes become integrated into post-transcriptional regulatory networks.

Our integrated analysis of gene transcription and m6A methylation revealed no direct relationship between the two, emphasizing m6A’s role as a modulatory layer in gene regulation. A focused examination of two groups of genes with high transcription and significant methylation levels uncovered complex, gene-specific regulatory patterns, highlighting the intricate interplay between m6A modification and gene transcription. The varying trajectories of genes like *NTH1, IME1*, and *SW16* in PCGs, and *YCL058C, YCR087W, and YKR035C* in DNGs, suggest that m6A methylation plays a critical, yet context-dependent role in regulating RNA stability and transcription during sporulation. These findings support the hypothesis that m6A methylation is not a uniform modification but instead operates in a nuanced manner to ensure the timely and coordinated transcription of meiotic regulators. Research has demonstrated that m6A methylation primarily influences RNA fate after transcription, impacting processes such as RNA stability and translation efficiency, rather than directly altering transcription rates (Agarwala et al., 2012; Schwartz et al., 2013; Fitzsimmons and Batista, 2019; Zhang et al., 2021). The implications of our findings extend beyond yeast sporulation, offering insights into the broader evolutionary context of gene regulation. This raises the possibility that the epitranscriptomic profile of a young gene is shaped first by its physical and sequence characteristics, with regulatory integration likely following as these features accumulate, a sequence of events that future functional studies could resolve.

While our study provides valuable insights into the m6A landscape of DNGs and PCGs during yeast sporulation, it is not without limitations. The relatively small sample size of identified DNGs compared to PCGs presents statistical challenges that could affect comparative analysis robustness. To mitigate these limitations, we implemented multiple complementary strategies, including length and expression matched PCG controls, permutation testing with 1,000 iterations, partial correlation and multivariable OLS regression to disentangle the effects of transcript length, expression level, and evolutionary age, length normalized motif density (per 100 nt), and appropriate multiple testing corrections. Together, these approaches support robust interpretation of the results despite inherent sample size constraints. Additionally, our analysis is based on correlative observations, and further experimental validation would be necessary to establish causal relationships between m6A modifications and gene regulation in DNGs. Future research should focus on elucidating the precise mechanisms by which m6A modifications influence the regulation and evolution of DNGs. Comparative studies across different species and developmental processes could provide a more comprehensive understanding of the evolutionary trajectory of m6A modifications in newly emerged genes. Furthermore, targeted manipulation of m6A sites in DNGs could offer insights into their functional significance and potential role in adaptive evolution.

## 5 Conclusion

This study provides novel insights into the m6A modification landscape of *de novo* genes (DNGs) relative to established protein-coding genes (PCGs) during yeast sporulation. Our findings reveal that DNGs exhibit a lower apparent per-gene m6A level than PCGs, accompanied by fewer peaks, reduced motif enrichment and density, and distinct patterns of m6A distribution across transcripts. Importantly, this reduced m6A level is substantially influenced by the shorter transcript length and lower expression of DNGs. Multivariable analysis indicated that GAC motif density, rather than evolutionary age, is the principal determinant of m6A deposition. The independent contribution of evolutionary age appears modest and is not statistically supported once sequence and length features are accounted for. Both gene classes nonetheless display dynamic, stage-specific m6A changes across sporulation, reflecting their shared regulation during meiotic progression. Together, these observations support the role of m6A as a context-dependent post-transcriptional regulatory layer, while indicating that the lower m6A modification of DNGs primarily reflects their structural and sequence characteristics rather than evolutionary age acting independently. Whether DNGs progressively acquire m6A regulation as a function of maturation remains an open question requiring further validation. Future research should focus on elucidating the precise mechanisms by which m6A modifications influence the regulation and evolution of DNGs across different species and developmental processes, potentially offering insights into their role in adaptive evolution and phenotypic innovation.

## Data Availability

The original contributions presented in the study are included in the article/Supplementary Material, further inquiries can be directed to the corresponding authors.
