# Supplementary material for: Comparative analysis of m6A mRNA modification in de novo and established genes during yeast sporulation
Source: Front Genet. 2026 Jul 29;17:1856601. doi: 10.3389/fgene.2026.1856601 (PMC13461074; doi:10.3389/fgene.2026.1856601)
Supplement: Supplementary file 1 [file Supplementaryfile1.docx]

Supplementary Figures

Comparative Analysis of m6A mRNA Modification in De Novo and Established Genes During Yeast Sporulation

Authors:

Azka Saleem¹, Yingshan Jiang¹, Jian Xue¹, Yuxuan Cui¹, Chuli Zhou¹, Fu Liu¹, Yuan Liu¹, Yubin Yan¹*, Ruolin Yang¹*

*¹ College of Life Sciences, Northwest A&F University, Yangling, Shaanxi, 712100, China*

***Author for Correspondence:**

**Yubin Yan**: [yanyb@nwafu.edu.cn](mailto:yanyb@nwafu.edu.cn)

**Ruolin Yang**: [desert.ruolin@gmail.com](mailto:desert.ruolin@gmail.com)

**Figure S1. Comparison of S288C reference genome selection over SK1 for m6A analysis.** (A) De novo genes (DNGs) annotation coverage between S288C and SK1 reference genomes across the primary (84) and secondary (213) DNGs sets. (B) HISAT2 alignment rates for all Input and IP samples aligned to S288C and SK1, with mean alignment rates of 85.7% and 74.5%, respectively. (C) Number of unique genes with at least one m6A peak detected in the S288C and SK1 genomes at each sporulation timepoint (T1, T3, T5, T7, T9, and PC), based on exomePeak2 results. (D) Pearson correlation coefficient *(r)* of mean log2 fold change (log2FC) values between SK1 and S288C derived results for shared genes across all timepoints, with DNGs (84: red, 213: orange) and protein coding genes (PCGs, gray) shown separately. (E) Pearson’s *r* of peak scores between the two genomes across all timepoints, confirming near-identical peak quality for shared genes. For all panels, teal represents SK1 and gold represents S288C.

**Figure S2. Distribution of m6A peak counts in PCGs and DNGs.** The histogram shows the significant difference in m6A peak abundance between PCGs and DNGs. PCGs display consistently higher proportions of transcripts with multiple m6A peaks (2 or ≥3), while DNGs are overrepresented in the zero-peak category. For statistical robustness, PCGs were down sampled 100 times to match DNG sample size, with results presented as mean ± standard deviation percentages.

**Figure S3. Comparative methylation abundance between expressed and translated DNGs.** Percentage of genes with varying numbers of m6A peaks (0, 1, 2, or ≥3 peaks) across different time points in split set expressed and translated DNGs.

 **Figure S4. Comparison of m6A methylation patterns across protein-coding genes (PCGs), de novo genes (DNGs).** Percentage of genes with varying numbers of m6A peaks (0, 1, 2, or ≥3 peaks) across different time points in PCGs and 213 DNG transcripts of *Blevins et al*.

**Figure S5. Comparative methylation abundance between expressed and translated DNGs.** Percentage of genes with varying numbers of m6A peaks (0, 1, 2, or ≥3 peaks) across different time points in split set expressed and translated DNGs.

**Figure S6. Relationship between m6A levels and gene age**. (A) m6A levels measured as peak scores across gene age groups (I&II, III, WGD, IV, V) at different sporulation timepoints, showing consistent patterns of decreasing methylation in younger gene groups. (B) Gene expression levels (TPM) across the same age classification groups and timepoints, demonstrating parallel expression patterns correlated with evolutionary age. (C) Gene length distribution (bp) across the five age categories. Age classification follows, Group I&II contains ancient genes conserved to filamentous fungi (>325 mya) and Ascomycota, Group III includes genes conserved to Saccharomycetaceae, WGD comprises duplicate genes from whole-genome duplication, Group IV contains genes conserved to Saccharomyces genus, and Group V comprises the youngest species-specific genes including de novo genes.

 **Figure S7.** **Length-Stratified Metagene Profiles of m6A Peaks in PCGs.** (A) Metagene profiles of m6A peak frequency across 72 length- and expression-matched PCGs (median length 372 bp; matched to DNG distribution median 378 bp) across all sporulation timepoints. (B) Metagene profiles of m6A peak frequency across all PCGs stratified into four transcript length quartiles: Q1 (51–567 bp, n=1,620, includes DNG length range), Q2 (570–1,092 bp, n=1,618), Q3 (1,095–1,785 bp, n=1,612), and Q4 (1,788–14,733 bp, n=1,612), across all timepoints (T1–PC). Gene body positions are scaled to equal length (TSS−20% to TTS+20%, 80 bins); dashed lines mark TSS and TTS. Shaded ribbons represent 95% bootstrap confidence intervals. Y-axis limits are consistent across all panels.

**Figure S8.** **Representative single-gene log2(IP/Input) coverage tracks illustrating per-gene heterogeneity in m6A enrichment topology.** (A) Three representative PCGs (YGL068W, YOR307C, YGL192W) and (B) three representative DNGs (YDR327W, YGR011W, YDL158C), shown across all sporulation timepoints (T1–PC). Pink regions indicate log2(IP/Input) > 0 (IP enriched), blue regions indicate log2(IP/Input) < 0 (IP depleted), with the signal curve overlaid. X-axis shows genomic coordinates (bp); strand direction indicated by arrow below the gene body bar. Individual genes display diverse enrichment/depletion patterns and temporal dynamics that are not captured by population-level metagene profiles (Figure 2A, B), reflecting gene-to-gene heterogeneity in m6A topology**.**

**Figure S9. Dynamic m6A modifications during sporulation timepoints.** (A, B) Temporal changes in m6A modifications via Differential Log2FC between adjacent timepoints for PCGs and DNGs, respectively. Error bars represent the minimum and maximum values, excluding outliers, for each timepoint.
